# Supplementary material for: The Evolution and Characterization of the RNA Interference Pathways in Lophotrochozoa
Source: Genome Biol Evol. 2024 May 7;16(5):evae098. doi: 10.1093/gbe/evae098 (PMC11114477; doi:10.1093/gbe/evae098)
Supplement: evae098_Supplementary_Data [file evae098_supplementary_data.zip › Supplementary_materials.zip!/selected_miRNAs/CrGi_selected_miRNAs/CrGi_NC_047560.1_7536.pdf]

Provisional ID	: NC_047560.1_7536
Score total	: 509.1
Score for star read(s)	: 3.9
Score for read counts	: 502.8
Score for mfe	: 1.4
Score for randfold	: 1.6
Score for cons. seed	: -0.6
Total read count	: 998
Mature read count	: 990
Loop read count	: 0
Star read count	: 8

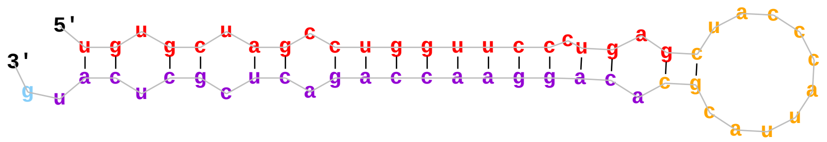

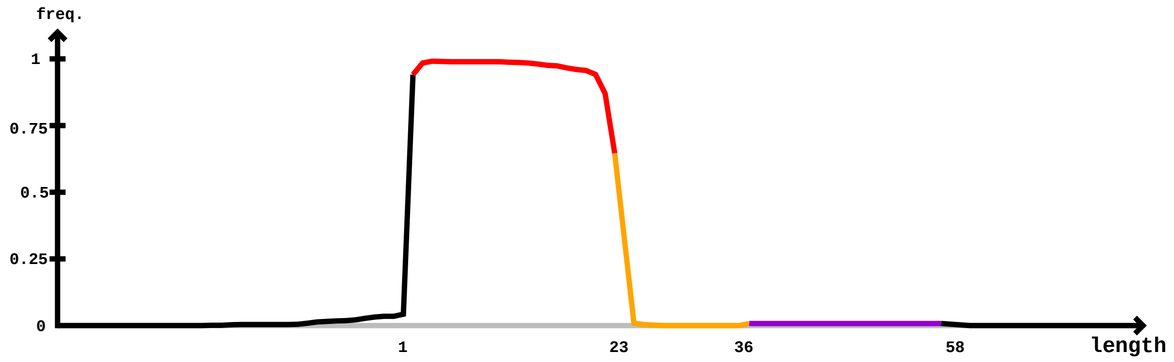

Mature Star

5'-	agggcauggaaucccgaucccgauucagauaaacgugugcuagccugguucccugagcuacccauuacgcacaggaaccagacucgcucaugcgcagacugaauuuucccc	-3'	obs		
	agggcauggaaucccgaucccgauucagauaaacgugugcuagccugguucccugagcuacccauuacgcacaggaaccagacucgcucaugcgcagacugaauuuucccc		exp		
	.(((..(((....)))..)))(((((((.....((((.((.((.((((((((.((.((..........)).)))))))))).)).)).))))......)))))))......		reads	mm	sample
	.................ucccgauucagauaGacgugugcuagcc..		1	1	Mt1
	........................ucagauaaacgugugcuagccugguu...		1	0	Mt1
	........................ucagauaaacgugugcuagccugguuc..		1	0	Mt1
	.........................cagauaaacUugugcuagccugg...		1	1	Mt1
	...........................gauaaacgugugcuagccug..		1	0	Mt1
	.............................uaaacgugugcuagccugg...		1	0	Mt1
	.............................uGaacgugugcuagccugguucccugag..		1	1	Mt1
	..............................aaacgugugcuagAcuggu..		1	1	Mt1
	..............................aaacgugugcuagccugguuc..		2	0	Mt1
	...............................aacgugugcuagccugguucc...		2	0	Mt1
	...............................aacgugAgcuagccugguucccugagcuac..		1	1	Mt1
	..................................gugugcuagccugguucccug..		1	0	Mt1
	..................................Uugugcuagccugguucccug..		2	1	Mt1
	..................................Uugugcuagccugguucccugag..		1	1	Mt1
	...................................ugugcuagccugguucccu...		6	0	Mt1
	...................................ugugcuagccCgguucccug..		1	1	Mt1
	...................................ugugcuGgccugguucccug..		1	1	Mt1
	...................................ugugcuagccugguucccug..		13	0	Mt1
	...................................ugugcuagccugguucccuga...		57	0	Mt1
	...................................ugugcuagccugguuGccuga...		1	1	Mt1
	...................................ugugcuaAccugguucccuga...		1	1	Mt1
	...................................ugugcuagccugguucccugag..		158	0	Mt1
	...................................ugugcuagccuAguucccugag..		2	1	Mt1
	...................................ugugcuaAccugguucccugag..		1	1	Mt1
	...................................ugugcuagccugguucccugGg..		1	1	Mt1
	...................................ugugcuagccCgguucccugag..		1	1	Mt1
	...................................ugugcuagccugguucccGgag..		1	1	Mt1
	...................................ugugcuUgccugguucccugag..		1	1	Mt1
	...................................ugugcuagccugguucccugagc...		9	0	Mt1
	...................................ugugcuagccugguucccugagcu..		1	0	Mt1
	...................................ugugcuagccugguucccugagcuaA..		1	1	Mt1
	....................................gugcuagccugguucccuga...		2	0	Mt1
	....................................gugcuagccugguucccugag..		4	0	Mt1

Mature **Star**

```
agggcauggaaucccgaucccgauucagauaaacgugugcuagccugguucccugagcuacccauuacgcacaggaaccagacucgcucaugcgcagacugaauuuucccc
....................................gugcuagccugguucccugagc.....................................................   3     0     Mt1
....................................gugcuagccugguucccugagcu....................................................   1     0     Mt1
.....................................ugcuagccugguucccug........................................................   1     0     Mt1
.....................................ugcuagccugguucccugag......................................................   1     0     Mt1
.....................................ugcuagccugguucccugagc.....................................................   1     0     Mt1

..............cgaucccgauucagauaaacgugu.........................................................................   1     0     Mt2
................aucccgauucagauaaacguA..........................................................................   1     1     Mt2
.......................uucagauaaacgugugcuagccu.................................................................   1     0     Mt2
........................ucagauaaacUugugcuagccugg...............................................................   1     1     Mt2
........................ucagauaaacgugugUuagccuggu..............................................................   1     1     Mt2
..........................agauaaaUgugugcuagccugguucc...........................................................   1     1     Mt2
..............................aaacgugugcuagAcugguu.............................................................   1     1     Mt2
..............................aaacgugugcuagAcugguuc............................................................   1     1     Mt2
................................acgugugcuagccugguucc...........................................................   1     0     Mt2
..................................gugugcuagccugguucccug........................................................   1     0     Mt2
...................................ugugcuagccugguuccc..........................................................   1     0     Mt2
...................................ugugcuagccugguuccU..........................................................   1     1     Mt2
...................................ugugcuagccugguucccu.........................................................   3     0     Mt2
...................................ugugcuagccugguucccug........................................................   13    0     Mt2
...................................ugugcuagccugguucccuga.......................................................   53    0     Mt2
...................................ugugcuagccugguucccuCa.......................................................   1     1     Mt2
...................................ugugcuaAccugguucccuga.......................................................   1     1     Mt2
...................................ugugcuagccCgguucccuga.......................................................   1     1     Mt2
...................................uguUcuagccugguucccugag......................................................   1     1     Mt2
...................................ugugcuagccugguucccugCg......................................................   1     1     Mt2
...................................ugugcuagccugguucAcugag......................................................   1     1     Mt2
...................................ugugcuagccugguucccugag......................................................   136   0     Mt2
...................................ugugcuagccCgguucccugag......................................................   2     1     Mt2
...................................ugugcuaAccugguucccugag......................................................   2     1     Mt2
...................................uguUcuagccugguucccugagc.....................................................   1     1     Mt2
...................................ugugcuagccugguucccugagc.....................................................   4     0     Mt2
...................................ugugcuagccCgguucccugagc.....................................................   1     1     Mt2
....................................gugcuagccugguucccug........................................................   1     0     Mt2
....................................gugcuagccugguucccuga.......................................................   1     0     Mt2
....................................gugcuagccugguucccugCg......................................................   1     1     Mt2
....................................gugcuagccugguucccugag......................................................   7     0     Mt2
....................................gugcuagccugguucccugagc.....................................................   4     0     Mt2
....................................gugcuagccugguucccugagcu....................................................   2     0     Mt2
......................................................................acaggaaccagacucgcucau....................   3     0     Mt2
......................................................................acaggaaccagacucgcucaug...................   1     0     Mt2

.........................cagauaaacgugugcuagcc..................................................................   1     0     Ov2
.........................cagauaaacgugugcuagAcug................................................................   1     1     Ov2
.........................cagauaaacgugugcuagccugg...............................................................   1     0     Ov2
..........................agauaaacgugugcuGgccuggu..............................................................   1     1     Ov2
...........................gauaaacgugugcuagccuggu..............................................................   1     0     Ov2
...............................aacgugugcuagccugguuc............................................................   1     0     Ov2
...............................aacgugugcuagccugguucc...........................................................   1     0     Ov2
..................................gugugcuagccugguuccc..........................................................   1     0     Ov2
..................................gCgugcuagccugguucccugagcua...................................................   1     1     Ov2
...................................ugugcuagccugguuccc..........................................................   1     0     Ov2
...................................ugugcuagUcugguucccu.........................................................   1     1     Ov2
...................................ugugcuagccugguucccu.........................................................   4     0     Ov2
...................................ugugcuagccugguucAcug........................................................   1     1     Ov2
...................................ugugcuagccugguucccug........................................................   16    0     Ov2
...................................Agugcuagccugguucccuga.......................................................   1     1     Ov2
...................................uguUcuagccugguucccuga.......................................................   1     1     Ov2
...................................ugugcuagccugguucccuga.......................................................   51    0     Ov2
...................................ugugcuagccCgguucccuga.......................................................   3     1     Ov2
...................................Cgugcuagccugguucccugag......................................................   1     1     Ov2
...................................ugugcuagccugguucccugCg......................................................   2     1     Ov2
...................................ugugcuGgccugguucccugag......................................................   2     1     Ov2
...................................ugugcuaAccugguucccugag......................................................   2     1     Ov2
...................................ugugcuagccugguucccugag......................................................   139   0     Ov2
...................................Ggugcuagccugguucccugag......................................................   1     1     Ov2
...................................ugugcuagccCgguucccugag......................................................   1     1     Ov2
...................................ugugcuagccugguucccugagc.....................................................   6     0     Ov2
...................................ugugcuaAccugguucccugagc.....................................................   1     1     Ov2
....................................gugcuagccugguucccug........................................................   1     0     Ov2
```

Mature Star

agggcauggaaucccgaucccgauucagauaaacgugugcuagccugguucccugagcuacccauuacgcacaggaaccagacucgcucaugcgcagacugaauuuucccc			
....................................gugcuagccugguucccugag..	5	0	Ov2
....................................gugcuagccugguucccugagc...	2	0	Ov2
....................................gugcuagccugAuucccugagc...	1	1	Ov2
....................................gugcuagccugguucccugagcu..	1	0	Ov2
....................................gugcuagccugguucccugagcuU...	1	1	Ov2
.....................................ugcuagccugguucccuga...	2	0	Ov2
.....................................ugcuagccugguucccugag..	1	0	Ov2
....acaggaaccagacucgcucau....................	3	0	Ov2
................aucccgauucagauaaacgugug..	1	0	Ov1
.........................cagauaaacgugugcuUgccugguucc...	1	1	Ov1
............................auaaacgugugcuagccuggu..	1	0	Ov1
.............................uaaacgugugcuagccugguucccug..	1	0	Ov1
..............................aaacgCgugcuagccugguuc..	1	1	Ov1
................................acUugugcuagccugguuc..	1	1	Ov1
................................acgugugcuagccugguuc..	1	0	Ov1
..................................Uugugcuagccugguucccugag..	1	1	Ov1
...................................ugugcuagccugguucccu...	1	0	Ov1
...................................ugugcuagccugguucccCg..	1	1	Ov1
...................................ugugcuagccugguucccug..	19	0	Ov1
...................................uguUcuagccugguucccuga...	1	1	Ov1
...................................ugugcuagccugguucccuga...	52	0	Ov1
...................................ugugcuaAccugguucccuga...	1	1	Ov1
...................................ugugcuagccCgguucccuga...	1	1	Ov1
...................................ugugcuagccugguucUcugag..	1	1	Ov1
...................................ugugcuaAccugguucccugag..	2	1	Ov1
...................................ugugcuGgccugguucccugag..	1	1	Ov1
...................................ugugcuagccCgguucccugag..	2	1	Ov1
...................................ugugcuagccugguucccugCg..	1	1	Ov1
...................................Cgugcuagccugguucccugag..	1	1	Ov1
...................................ugugcuagccuAguucccugag..	2	1	Ov1
...................................ugugcuagccugguucccugag..	117	0	Ov1
...................................Ggugcuagccugguucccugag..	1	1	Ov1
...................................ugugcuagccAgguucccugagc...	1	1	Ov1
...................................ugugcuagccugguucccugagU...	2	1	Ov1
...................................ugugcuagccCgguucccugagc...	2	1	Ov1
...................................ugugcuagccugguucccugagc...	7	0	Ov1
....................................gugcuagccugguucccug..	1	0	Ov1
....................................gugcuagccugguucccugag..	4	0	Ov1
....................................gugcuagccugguucccugagc...	2	0	Ov1
....................................gugcuagccugguucAcugagc...	1	1	Ov1
.....................................ugcuagccugguucccuga...	1	0	Ov1
.....................................ugcuagccugguucccugag..	1	0	Ov1
....acaggaaccagacucgcucau....................	1	0	Ov1
